# Discovery and validation of an NMR-based metabolomic profile in urine as TB biomarker

**DOI:** 10.1038/s41598-020-78999-4

**Published:** 2020-12-18

**Authors:** José Luis Izquierdo-Garcia, Patricia Comella-del-Barrio, Ramón Campos-Olivas, Raquel Villar-Hernández, Cristina Prat-Aymerich, Maria Luiza De Souza-Galvão, Maria Angeles Jiménez-Fuentes, Juan Ruiz-Manzano, Zoran Stojanovic, Adela González, Mar Serra-Vidal, Esther García-García, Beatriz Muriel-Moreno, Joan Pau Millet, Israel Molina-Pinargote, Xavier Casas, Javier Santiago, Fina Sabriá, Carmen Martos, Christian Herzmann, Jesús Ruiz-Cabello, José Domínguez

**Affiliations:** 1 CIC biomaGUNE Center for Cooperative Research in Biomaterials, BRTA Basque Research and Technology Alliance, Donostia, Donostia, Gipuzkoa Spain; 2 CIBER de enfermedades respiratorias (CIBERES), Instituto de Salud Carlos III, Madrid, Spain; 3 Facultad de Farmacia, Universidad Complutense de Madrid, Madrid, Spain; 4 Servei de Microbiologia, Hospital Universitari Germans Trias i Pujol, Institut d’Investigació Germans Trias i Pujol, Badalona, Barcelona Spain; 5 Departament de Genètica i Microbiologia, Universitat Autònoma de Barcelona, Barcelona, Spain; 6 CNIO Centro Nacional de Investigaciones Oncológicas, Madrid, Spain; 7 Unitat de Tuberculosi de Drassanes, Servei de Pneumologia, Hospital Universitari Vall d’Hebron, Barcelona, Spain; 8 Servei de Pneumologia, Hospital Universitari Germans Trias i Pujol, Barcelona, Spain; 9 Serveis Clínics, Unitat Clínica de Tractament Directament Observat de la Tuberculosi, Barcelona, Spain; 10 CIBER de Epidemiología y Salud Pública (CIBERESP), Instituto de Salud Carlos III, Madrid, Spain; 11 Servei de Pneumologia, Hospital Sant Joan Despí Moises Broggi, Sant Joan Despi, Barcelona Spain; 12 Center for Clinical Studies, Research Center Borstel, Borstel, Germany; 13 IKERBASQUE, Basque Foundation for Science, Bilbao, Vizcaya Spain; 14 Present Address: Julius Centre for Health Sciences and Primary Care, University Medical Center Utrecht, Utrecht University, Utrecht, The Netherlands

**Keywords:** Biomarkers, Tuberculosis, Biological models, Metabolomics

## Abstract

Despite efforts to improve tuberculosis (TB) detection, limitations in access, quality and timeliness of diagnostic services in low- and middle-income countries are challenging for current TB diagnostics. This study aimed to identify and characterise a metabolic profile of TB in urine by high-field nuclear magnetic resonance (NMR) spectrometry and assess whether the TB metabolic profile is also detected by a low-field benchtop NMR spectrometer. We included 189 patients with tuberculosis, 42 patients with pneumococcal pneumonia, 61 individuals infected with latent tuberculosis and 40 uninfected individuals. We acquired the urine spectra from high and low-field NMR. We characterised a TB metabolic fingerprint from the Principal Component Analysis. We developed a classification model from the Partial Least Squares-Discriminant Analysis and evaluated its performance. We identified a metabolic fingerprint of 31 chemical shift regions assigned to eight metabolites (aminoadipic acid, citrate, creatine, creatinine, glucose, mannitol, phenylalanine, and hippurate). The model developed using low-field NMR urine spectra correctly classified 87.32%, 85.21% and 100% of the TB patients compared to pneumococcal pneumonia patients, LTBI and uninfected individuals, respectively. The model validation correctly classified 84.10% of the TB patients. We have identified and characterised a metabolic profile of TB in urine from a high-field NMR spectrometer and have also detected it using a low-field benchtop NMR spectrometer. The models developed from the metabolic profile of TB identified by both NMR technologies were able to discriminate TB patients from the rest of the study groups and the results were not influenced by anti-TB treatment or TB location. This provides a new approach in the search for possible biomarkers for the diagnosis of TB.

## Introduction

Tuberculosis (TB) is the leading cause of death by infectious disease worldwide. Despite efforts to improve TB detection through advances in diagnosis and accessibility to treatment, there are still more than 2–3 million unidentified TB cases at the moment. Misdiagnosis and late detection of the disease increase the risk of *Mycobacterium tuberculosis* transmission and infection. Progress in controlling TB and mitigating its consequences can be expedited through early diagnosis and treatment^1^.

Among current diagnostics, culture is the gold standard for diagnosing TB, but it takes approximately 4–6 weeks to obtain results^2^. Therefore, in low-income countries where the burden of disease is high, smear microscopy and X-ray are the main tests used. However, these have shown limited diagnostic sensitivity and specificity, respectively^3,4^. The introduction of Xpert MTB/RIF (Cepheid, CA, USA) as a molecular method for TB diagnosis has considerably improved the time of diagnosis and detection of resistance to treatment, its use has increased as an alternative to culture and smear microscopy^5^. However, cost and infrastructure requirements prohibit its implantation and use in most microscopy centres^6^. In addition, its poor performance when testing individuals with low bacilli numbers (children, HIV-co-infected patients, extrapulmonary TB cases, early stages of disease), require consumables that are expensive or locally unavailable due to stock-outs^7^. Limitations of accessibility, quality and timing of diagnostic services in low and middle-income countries (LMICs) represent a challenge for current TB diagnostics. Future research should be focused on developing an accurate and rapid biomarker-based test that can diagnose all forms of TB using non-sputum samples, ideally one suitable for use in both primary healthcare centres and regional centres^8^.

Metabolomics has emerged from the ‘omics’ technologies as a tool to obtain a fingerprint of all the metabolites present in a cellular system, allowing discrimination between samples with a different biological status^9^. In this approach, metabolomics has been applied to study the metabolites affected by host–pathogen interactions and identify diagnostic markers to improve diagnosis of different respiratory infectious diseases^10^. In recent years, metabolomic studies have been conducted to gain novel biological insights into TB pathogenesis^11^. Thus, metabolomics has been used to study TB progression and detect metabolic profiles, as well as to assess TB treatment response from different biological specimens^12^. Urine is an abundant sterile, biological sample that is obtained non-invasively and requires little preparation^13^. However, few metabolomic studies focus on the discovery of new urine-based biomarkers for TB detection. Metabolic changes in any type of sample can be measured through different analytical techniques summarised in mass spectrometry (MS) and Nuclear Magnetic Resonance (NMR)^14^. Recently, a benchtop NMR spectrometer has been developed as a potential tool for point-of-care diagnostics in urine samples due to its high performance in a compact size^15,16^.

This study aimed to identify and characterise a metabolic profile of TB in urine by high-field NMR spectrometry and assess whether the TB metabolic profile is also detected by a low-field benchtop NMR spectrometer. The identification of a metabolic pattern for urine from an NMR technology would provide a new approach and advance in the search of potential biomarkers for TB diagnosis.

## Results

### Study population

Three hundred and thirty-two participants were included in this study and classified into the following study groups: 189 active TB patients, 42 pneumococcal pneumonia patients, 61 LTBI individuals, and 40 uninfected individuals. Demographics of the study population are shown in Table 1. Of the 332 patients, 64.8% were men of an average of 46 years old (± 17.3). Regarding study groups, most TB (untreated and under treatment) and pneumococcal pneumonia patients and individuals with LTBI were men (79.6%, 70.7%, 54.1%, and 81.0%, respectively), while 75.0% of the uninfected individuals were women. Patients with pneumococcal pneumonia were older than the rest of the study groups (49.6% older, p < 0.001). From the patients with active TB, 49 were enrolled before starting anti-TB treatment and 140 during TB treatment. Patients undergoing TB treatment averaged 39.1 (SD ± 68.9) days of treatment. Most patients (99.3%) had strains sensitive to TB treatment except one patient with rifampicin-resistant strains (Table 2). Among patients with TB, 78.8% had pulmonary TB, 13.8% had extrapulmonary TB (lymph nodal, pleural, peritoneal, osteoarticular, meningeal and miliary TB), and 7.4% had disseminated TB (Table 2).

**Table 1:** Demographic information of the study population.

Variable	All (n = 332)	TB untreated (n = 49)	TB under treatment (n = 140)	PnP (n = 42)	LTBI (n = 61)	Uninfected (n = 40)
**Gender**						
Feminine	117 (35.2%)	10 (20.4%)	41 (29.3%)	8 (19.0%)	28 (45.9%)	30 (75.0%)
Masculine	215 (64.8%)	39 (79.6%)	99 (70.7%)	34 (81.0%)	33 (54.1%)	10 (25.0%)
**Age in years**						
Media (SD)	45.9 (17.3)	43.0 (20.3)	43.5 (16.3)	64.4 (13.5)	42.6 (10.3)	43.1 (17.1)

Categorical variables expressed as a number of subjects (n) and percentage (%), and quantitative variables expressed as median and standard deviation (SD).

TB, tuberculosis; PnP, pneumococcal pneumonia; LTBI, latent TB infection; Uninfected, individuals without infection.

**Table 2:** Table describing the location and treatment of TB patients.

Variable	All (n = 189)	TB untreated (n = 49)	TB under treatment (n = 140)
**TB type**			
Pulmonary TB	149 (78.8%)	37 (75.5%)	112 (80.0%)
Extrapulmonary TB	26 (13.8%)	10 (20.4%)	16 (11.4%)
Disseminated TB	14 (7.4%)	2 (4.1%)	12 (8.6%)
**TB treatment**			
Drug-susceptible TB	186 (98.4%)	47 (95.9)	139 (99.3%)
RR-TB	1 (0.5%)	0 (0.0%)	1 (0.7%)
Hr-TB	1 (0.5%)	1 (2.0%)	0 (0.0%)
XDR-TB	1 (0.5%)	1 (2.0%)	0 (0.0%)

Variable expressed as a number of subjects (n) and percentage (%).

TB: tuberculosis; RR-TB: rifampicin-resistant TB; Hr-TB: isoniazid-resistant TB; XDR-TB: extensively drug-resistant TB.

**Figure 1:**
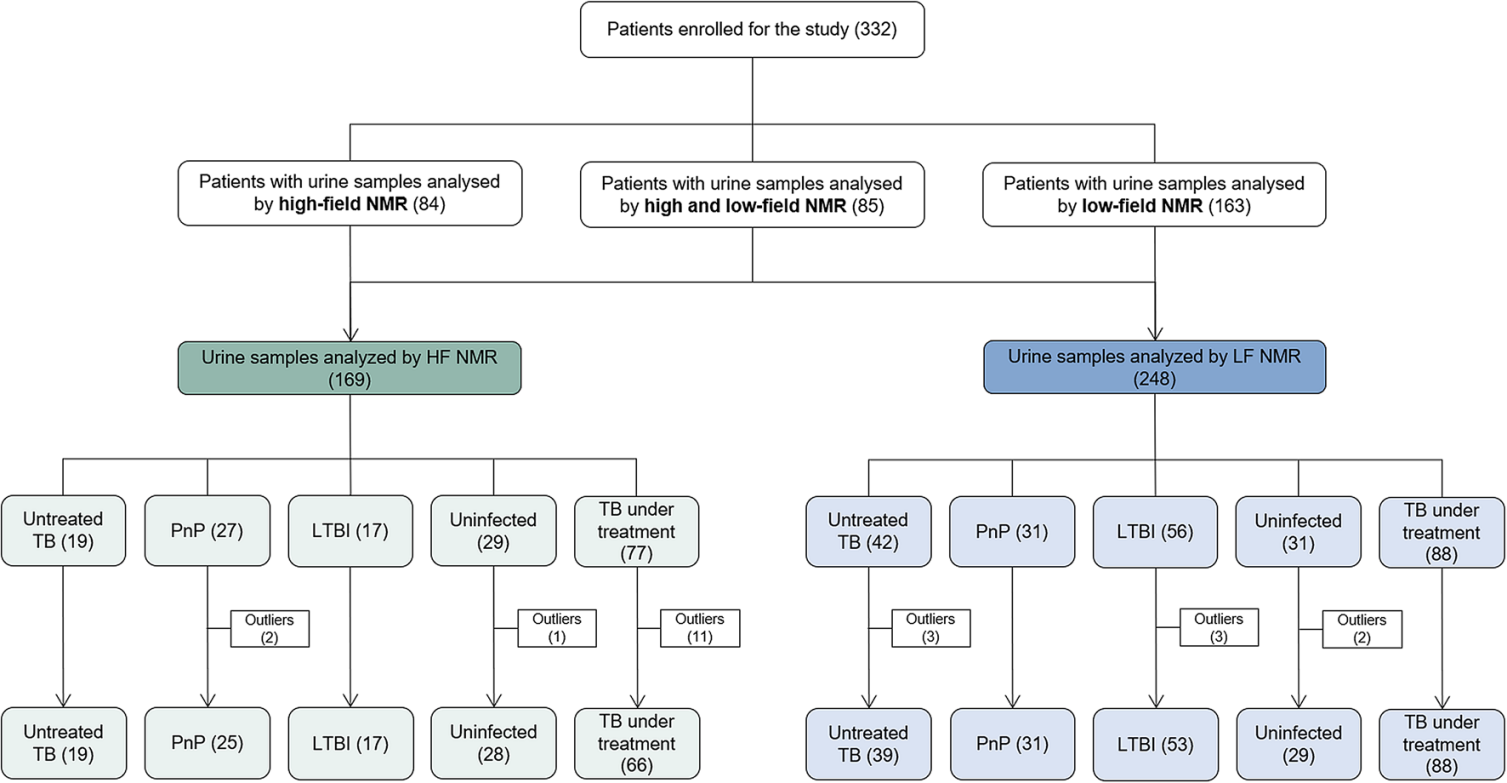
Description of the procedure followed to analyse the urine samples of the 332 participants by high and/or low NMR. NMR, Nuclear Magnetic Resonance; TB, tuberculosis; PnP, pneumococcal pneumonia; LTBI, latent TB infection; uninfected, individuals without infection.

Of the 332 urine samples obtained from the individual participants, 169 samples were analysed with HF-NMR to identify a metabolic profile that discriminated TB patients from study controls. Then, the remaining 163 samples plus 85 samples previously analysed by HF-NMR were analysed using an LF benchtop NMR spectrometer to detect the TB metabolic profile in a more compact device that can be installed in conventional laboratories and used as a diagnostic tool. The procedure followed to analyse urine samples by NMR is shown in Fig. 1.

### Characterisation of the TB metabolic fingerprint using HF-NMR

**Figure 2:**
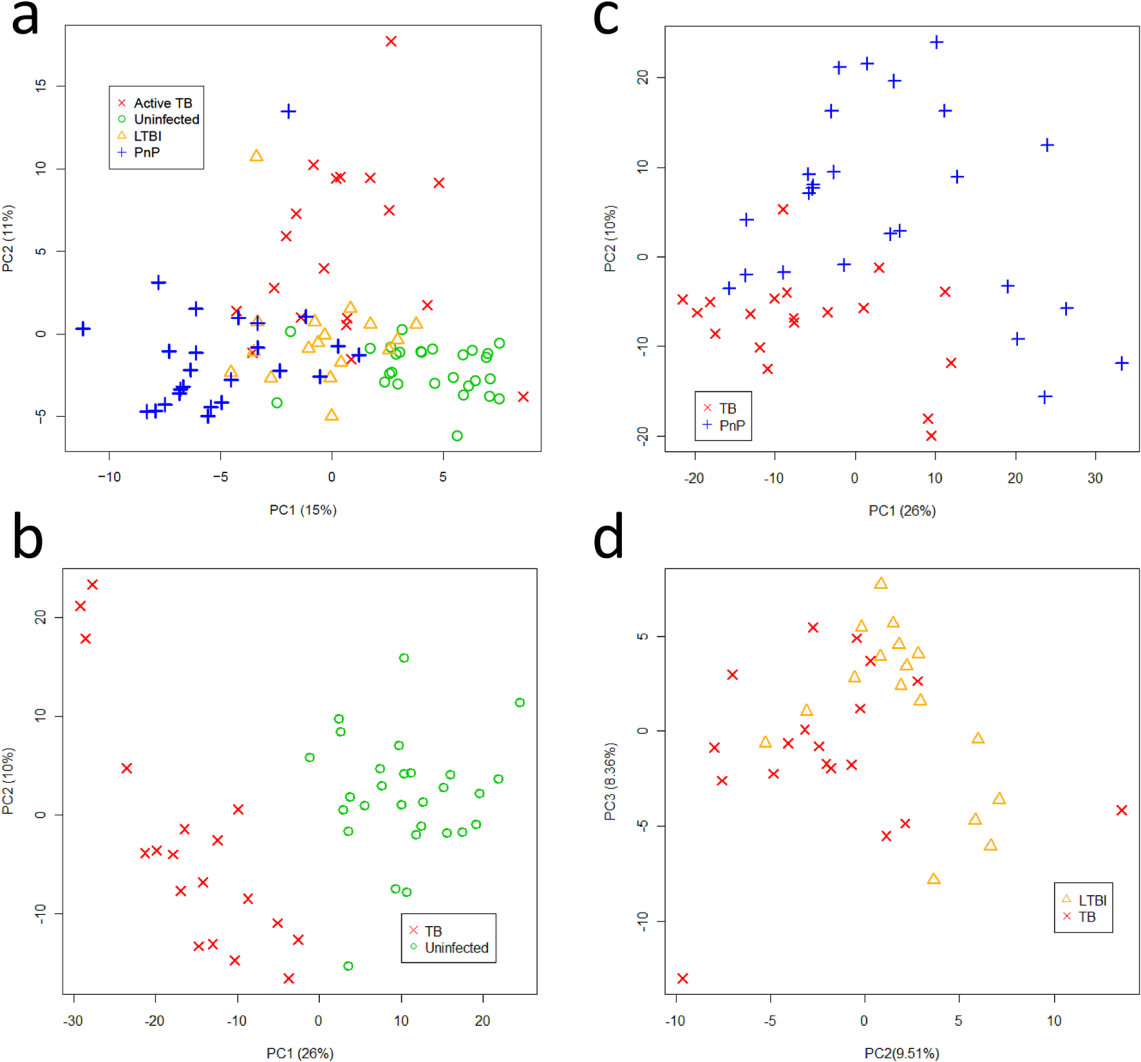
Principal Component Analysis (PCA) score plots of urine spectra analyzed by high-field Nuclear Magnetic Resonance of (**a**) untreated TB patients (n = 19), uninfected individuals (n = 28), pneumococcal pneumonia patients (n = 25) and LTBI individuals (n = 17); (**b**) untreated TB and uninfected individuals; (**c**) untreated TB and pneumococcal pneumonia patients; (**d**) untreated TB and LTBI individuals. TB, tuberculosis; PnP, pneumococcal pneumonia; LTBI: latent TB infection; PC, Principal Component.

**Figure 3:**
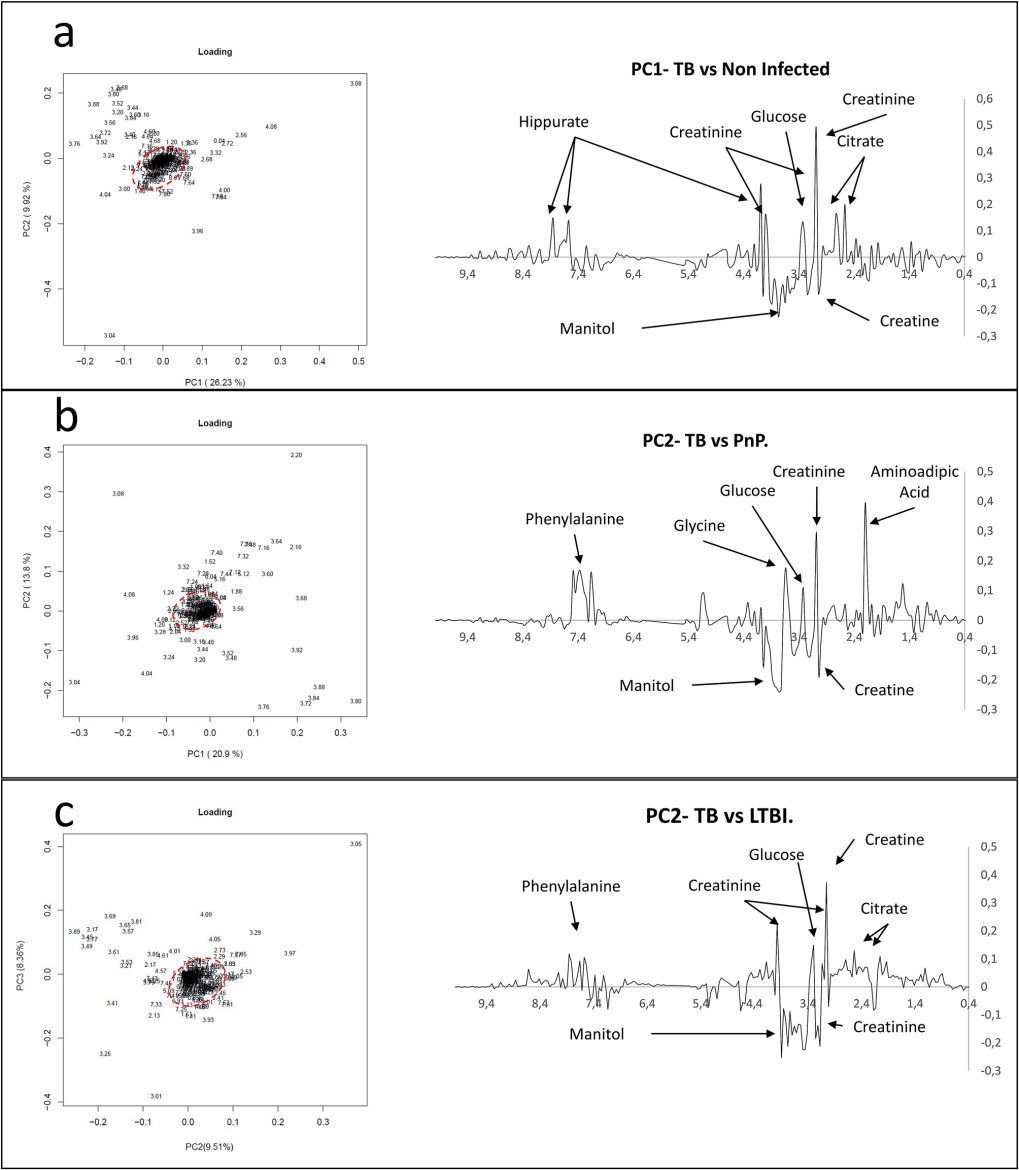
Principal Component Analysis (PCA) loading plots of 89 urine spectra analyzed by high-field Nuclear Magnetic Resonance reveals the metabolomic fingerprint of TB corresponding to 31 chemical shift regions assigned to eight metabolites. (**a**) PCA loading PC1-PC2 biplot and PC1 loading plot between TB patients and uninfected individuals; (**b**) PCA loading PC1-PC2 biplot and PC2 plot between TB patients and patients with pneumococcal pneumonia (PnP); (**c**) PCA loading PC2-PC3 biplot and PC2 loading plot between TB patients and individuals with LTBI. Multiple regions for the discrimination between groups were pointed outside the boundaries of a Hotelling's T2 statistics ellipse (pointed red line) in PCA loading biplots. TB, tuberculosis; PnP, pneumococcal pneumonia; LTBI: latent TB infection; PC, Principal Component.

We applied an unsupervised Principal Component Analysis (PCA) to the urine spectra acquired by HF-NMR (19 patients with untreated TB, 27 patients with pneumococcal pneumonia, 17 individuals with LTBI and 29 uninfected individuals) and detected 3 statistical outliers (2 patients with pneumococcal pneumonia and 1 uninfected individual) that were excluded from the analysis^17^. PCA score plots identified differential metabolic clusters between patients with untreated TB (n = 19) and pneumococcal pneumonia patients (n = 25), LTBI individuals (n = 17), and uninfected individuals (n = 28) (Fig. 2). The variability observed was not explained by the different participant recruitment centres (Supplementary Figure S1). The spectral regions responsible for the metabolic differences in each PCA scores plots between TB patients and control groups (pneumococcal pneumonia, LTBI, and uninfected) were identified in PCA loading plots by Hotteling’s T2 tests^18^ and correlated with a total of 31 chemical shift regions in the first two Principal Components (PCs) (Fig. 3). This urinary spectral fingerprint (corresponding to 31 spectral regions) was assigned to the following eight metabolites: aminoadipic acid, citrate, creatine, creatinine, glucose, mannitol, phenylalanine, and hippurate (Supplementary Figure S2). Metabolites were quantified to show the statistically substantial differences between study groups (Table 3).

**Table 3:** Relative change in the concentration of the identified metabolites.

Metabolite	Percentage change in concentration (%)			Bonferroni corrected T-test		
	TB vs. PnP	TB vs. LTBI	TB vs. Uninfected	TB vs. PnP	TB vs. LTBI	TB vs. uninfected
Aminoadipic	− 67.0	17.3	82.9	**0.0013**	0.6450	0.0975
Citrate	66.2	− 36.7	− 59.6	0.0881	**0.0162**	**0.0001**
Creatine	− 30.3	102.7	21.6	0.2690	**0.0407**	0.4470
Creatinine	91.6	− 19.4	− 32.3	**0.0012**	0.1790	**0.0013**
Glucose	37.8	− 24.6	− 7.9	0.3720	**0.0073**	0.7080
Mannitol	− 47.7	18.1	78.8	0.1070	0.2620	**0.0436**
Phenylalanine	− 33.4	60.4	33.5	**0.0262**	0.0321	0.0626
Hippurate	34.8	− 23.3	− 49.6	0.2700	0.4720	**0.0132**

TB, tuberculosis; PnP, pneumococcal pneumonia; LTBI, latent TB infection; uninfected, individuals without infection; PC: Principal Component.

Bold values, statistical significance was determined using a Bonferroni corrected Student's t-test assuming significant unequal corrected variance with p < 0.05.

### HF-NMR-based profile to discriminate TB

We applied a supervised Partial Least Squares-Discriminant Analysis (PLS-DA) to establish predictive models from the identified spectral fingerprint that differentiated untreated TB cases (n = 19) from control groups (25 pneumococcal pneumonia, 17 LTBI, and 28 uninfected). Thus, when comparing untreated TB and pneumococcal pneumonia patients, 100% of TB patients were correctly classified, with 100.0% of sensitivity and specificity. Similarly, when comparing TB patients and LTBI individuals, 94.0% (Standard Deviation, SD = 5.6%) of TB patients were correctly classified, with 89.4% (SD = 6.5%) and 93.9% (SD = 5.7%) sensitivity and specificity, respectively. Finally, when comparing TB patients and uninfected individuals, 100% of TB patients were correctly classified, with 100% of sensitivity and specificity, respectively.

We used the model established between untreated TB patients and uninfected individuals to classify the HF-NMR urine spectra of the remaining 66 TB patients (all of them under treatment). The model correctly classified 90.9% of these TB patients in the TB group. Among these TB patients, 51 had pulmonary TB, 11 had extrapulmonary TB, and 4 had disseminated TB. This model also correctly classified 90.9% of the extrapulmonary TB patients in the TB group.

### Characterisation of the TB metabolic profile using LF-NMR

**Figure 4:**
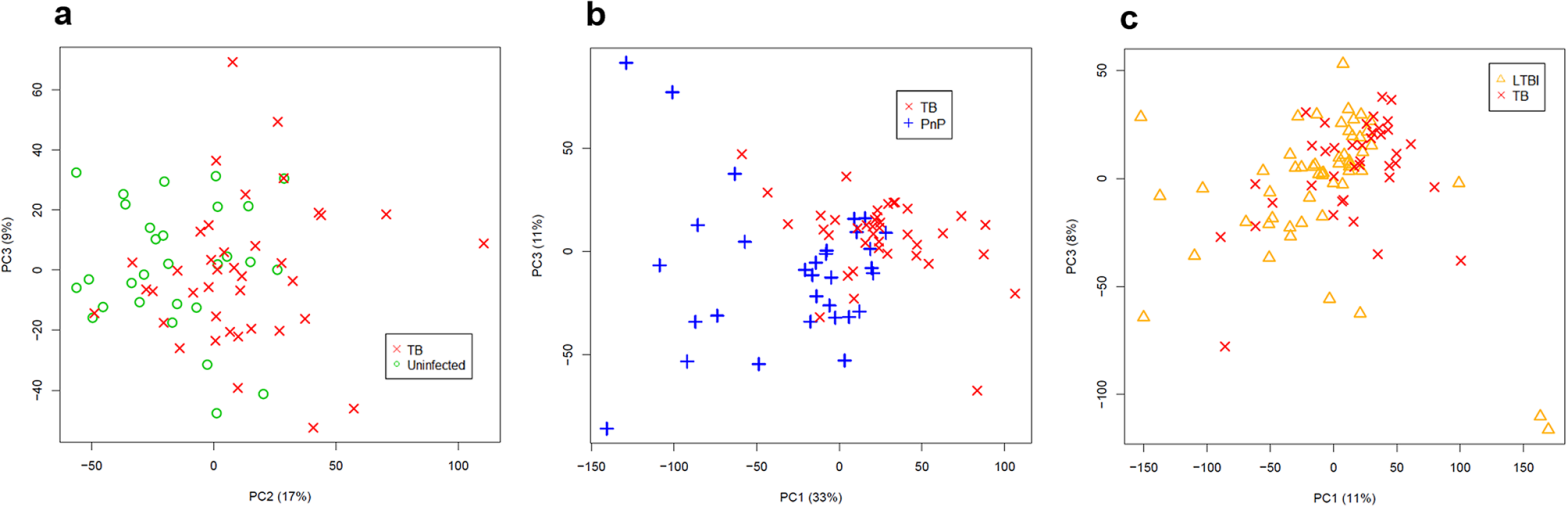
Principal Component Analysis (PCA) score plots of urine spectra analyzed by low-field Nuclear Magnetic Resonance between 39 untreated TB (x) and: (**a**) 29 uninfected individuals (circle), (**b**) 31 pneumococcal pneumonia patients (cross), and (**c**) 53 LTBI individuals (triangle). TB, tuberculosis; PnP, pneumococcal pneumonia; LTBI: latent TB infection; PC, Principal Component.

**Figure 5:**
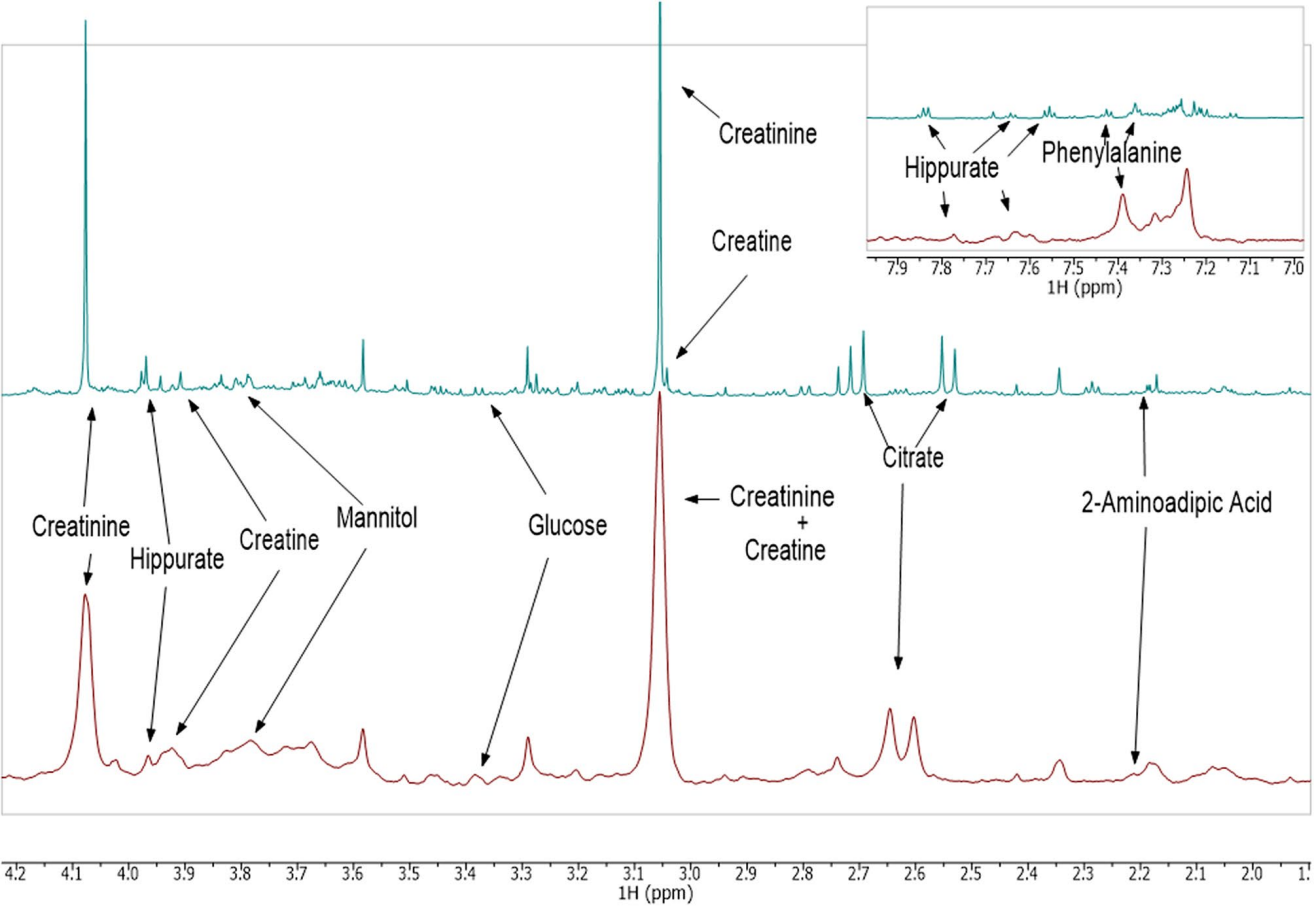
Comparison of the metabolomic fingerprints of tuberculosis (TB) identified in urine spectra analyzed by high-field Nuclear Magnetic Resonance (green) and low-field Nuclear Magnetic Resonance (red) showing the identification of the TB metabolite biomarkers. PC: Principal Component.

To detect the metabolic profile of TB in urine previously characterised using HF-NMR, we applied the unsupervised PCA to the urine spectra acquired by LF-NMR (42 patients with untreated TB, 31 patients with pneumococcal pneumonia, 56 individuals with LTBI and 31 uninfected individuals). We detected 8 statistical outliers (3 untreated TB, 3 LTBIs, and 2 uninfected), which were excluded from the analysis^17^. Thus, the unsupervised PCA was applied to a total of 39 untreated TB patients, 31 pneumococcal pneumonia patients, 53 LTBI individuals, and 29 uninfected individuals (Fig. 1). PCA score plots of the LF-NMR urine spectra did not show as clear a discrimination as the HF-data did (Fig. 4). However, although LF-NMR spectroscopy provides a lower resolution than HF-NMR spectroscopy, we identified the spectral fingerprint assigned to the eight metabolites (aminoadipic acid, citrate, creatine, creatinine, glucose, mannitol, phenylalanine, and hippurate) in the metabolic profile, which enabled the differentiation of patients with TB from the controls (Fig. 5).

### TB discrimination from LF-NMR-based metabolic profile

PLS-DA was applied to establish predictive models to discriminate untreated TB cases (n = 39) from control groups (31 pneumococcal pneumonia, 53 LTBI, and 29 uninfected) from the LF-NMR urinary spectral fingerprint identified. When comparing TB and pneumococcal pneumonia patients, 87.3% (SD = 7.8%) of TB patients were correctly classified, with 94.4% (SD = 3.6%) and 85.62% (SD = 5.5%) sensitivity and specificity, respectively. Similarly, when comparing TB patients and LTBI individuals, 85.2% (SD = 5.8%) of TB patients were correctly classified, with 91.9% (SD = 4.8%) and 90.2% (SD = 3.5%) sensitivity and specificity, respectively. Finally, when comparing TB and uninfected individuals, 100% of TB patients were correctly classified, with 100% of sensitivity and specificity.

The predictive model established between untreated TB patients and uninfected individuals was applied to classify the LF-NMR urine spectra of the 88 TB patients under treatment. The model correctly classified 84.1% of TB patients that had not been used to create the predictive model (all of them under treatment) in the TB group. Among these TB patients, 68 had pulmonary TB, 12 had extrapulmonary TB, and 8 had disseminated TB. This model also correctly classified 100% of extrapulmonary TB patients in the TB group.

## Discussion

In recent years a lot of effort has been made to identify the highest priority needs in order to improve the diagnostic procedures for TB^1,19^. The need to develop accurate and more accessible diagnostic methods in primary healthcare centres has meant an intensification of the search for biomarkers from non-sputum-based biological samples^20^.

In this study, we have identified and characterised a metabolic profile of TB in urine from a high-field NMR spectrometer and detected the same profile with a low-field NMR spectrometer. The models developed from the metabolic profile of TB identified by both NMR technologies showed the potential to discriminate between TB patients, pneumococcal pneumonia patients, individuals with LTBI and uninfected individuals. In addition, the results of the models developed from this metabolic fingerprinting were not influenced by anti-TB treatment or TB location.

When applying the predictive model to the HF and LF-NMR spectra of the TB patients under treatment, 90.9% and 84.1% of the patients in the TB group were correctly classified. Furthermore, the models developed from the HF and LF-NMR-based spectral fingerprints correctly classified TB patients and pneumococcal pneumonia patients with a success rate of 100% and 87.3%, respectively. Therefore, the use of the model developed here could facilitate the identification of patients with TB and rule out those with other respiratory infections, such as pneumococcal pneumonia.

Using the HF-NMR-based spectral fingerprint, our model correctly classified 94.0% of TB patients compared to LTBI individuals, and 85.2% of them when using the LF-NMR-based urine metabolic fingerprint. This may be useful in situations where it is necessary to distinguish between TB and LTBI, such as in children, where early detection of TB is crucial to avoid severe forms of the disease developing. It is well known that neither TST nor IGRAs can distinguish between LTBI and active cases^21,22^. In our experience^23^, a model based on the combination of IFN-γ, IP-10, ferritin and 25-hydroxyvitamin D could improve the detection of patients with subclinical TB. The metabolomic approach we described may be useful in detecting the early stages of the disease. Regarding metabolomics, a multi-site study across Sub-Saharan Africa provided a trans-African metabolic biosignature in serum and plasma to predict the onset of TB before active TB manifestation^24^.

The metabolomic approach we present showed a classification performance within the minimum standards required by the World Health Organization to develop a non-sputum based biomarker test^19^. Although Xpert for detecting TB is still not as sensitive as culture, its rapidity in diagnosing TB has made shortening the delay between diagnosis and early treatment possible^25^. Also considering speed, the ability of the LF benchtop NMR spectrometer to measure samples quickly and easily would enable the integration of the final decision on the diagnosis into the same visit.

In addition, the use of an easily accessible sample would allow for successful implementation in microscopy centres, health posts and primary-care clinics^26^. A urine-based approach would allow the diagnosis of TB in patients who had difficulty in obtaining a representative sample from the site of infection (patients with extrapulmonary TB or not able to produce sputum), a relevant issue especially in LMICs since these patients often present non-specific symptoms of the disease^1^. The Alere Determine TB LAM Ag assay (AlereLAM, Abbott, Chicago, US) was introduced for TB diagnosis in HIV-positive patients by detecting the presence of lipoarabinomannan (LAM) in urine samples^27^. Subsequently, a new generation of urine LAM assays, Fujifilm SILVAMP TB LAM (FujiLAM) assay (Fujifilm, Tokyo, Japan) has been developed^28^, representing improved diagnostics for HIV-positive TB patients. In this regard, the metabolic profile identified in this study might provide a promising tool for diagnosing TB from urine samples. In addition, the quantification of the statistically significant metabolites identified in this TB metabolic profile would allow this technology to be adapted to a point-of-care test. Although more studies should be conducted in larger TB cohorts, vulnerable populations (children, HIV, comorbidities), and in other geographic regions to validate the performance of the predictive model based on the metabolic profile identified by LF benchtop NMR technology, these first results are promising.

If we consider the target product profiles (TPPs) published by the WHO and partners^26^, although the diagnostic sensitivity of this metabolic model did not reach that achieved with the Xpert (diagnostic sensitivity of > 95% in comparison to culture), the sensitivity it achieved was within the minimum requirements established by the TPPs to develop a rapid non-sputum-based biomarker test for pulmonary TB in adults. Furthermore, with 100% of extrapulmonary TB patients correctly classified into the TB group, this model would fall within the optimal requirements recommended by the published TPPs for developing a rapid non-sputum-based biomarker test for extrapulmonary TB in adults^19^. Therefore, this metabolic approach based on LF-NMR could be a promising candidate for detecting all TB types. In addition, its small size and ease of maintenance would allow the implementation of the technology in primary healthcare centres as an alternative, or complementary to the diagnostic tests already available, without the risk of a shortage of cartridges. This robust benchtop NMR technology, straightforward sample preparation and minimal operational requirements would lead to a better diagnostic performance in LMICs; thus, increasing the number of cases diagnosed and allowing prompt treatment, which would reduce the transmission and mortality burden of TB.

TB is known to be a wasting disease involving weight loss, malnutrition and metabolic disorders^29^. The nutritional source of the bacteria is an essential aspect of host–pathogen interaction^24^. *M. tuberculosis* has adapted its metabolism to use different nutrient sources and compounds such as carbon or nitrogen sources to promote bacterial growth^30^. The metabolic profile presented in this study is based on the combination of eight metabolites, which is able to distinguish TB patients from those with pneumococcal pneumonia, LTBI, and non-infection. Previous studies have been conducted on serum samples to identify potential biomarkers for TB diagnosis using MS^31–33^ and NMR^34^. Others have identified TB diagnostic markers from plasma samples in adults^35,36^ and children^37^. In this study, TB patients showed reduced concentrations of creatine and phenylalanine compared to LTBI patients. Both metabolites are involved in the metabolism of necessary amino acids and derivatives. During infection in macrophages, *M. tuberculosis* shows a preference for amino acids as a source of nitrogen^38^. In line with this, TB patients showed high concentrations of creatinine compared to pneumococcal pneumonia patients. Creatinine is a breakdown product of creatine, so it might have a role in the synthesis of nitrogen-containing molecules. *M. tuberculosis* can co-metabolise multiple amino acids simultaneously^30^. In this metabolic process urea and other amino groups are synthesised from the breakdown of the amino acids. Thus, the low concentration of urine hippurate observed in TB patients might be due to/connected to the synthesis of aromatic amino acids such as tryptophan, tyrosine and phenylalanine^39,40^. Alternatively*, *Weiner et al. suggested that low serum hippurate concentrations might be related to uremic cytotoxic activity related to vitamin D metabolism^24^. In this study, TB patients showed low glucose and citrate concentrations compared with LTBI patients and uninfected individuals. The involvement of these two metabolites in the oxidative metabolism of carbohydrates, proteins and fats suggests that there is an increase in energy consumption by TB patients^41^. In accordance with this, Ehrt et al. reported the adaptation of *M. tuberculosis* to co-catabolise multiple carbon substrates, so that it grows faster and more extensively on carbon source mixtures than it does on any single source^30^. This might also explain the high concentrations of mannitol found in TB patients compared with non-infected individuals. However, in this study, we found that TB patients showed higher concentrations of citrate and lower concentrations of intermediary compounds of amino acid metabolism compared to pneumococcal pneumonia patients. This could be explained because the Krebs cycle is most likely not used in the *S. pneumoniae* metabolism due to the lack of genes encoding the enzymes involved in this pathway. In contrast, the lack of a complete Krebs cycle for *S. pneumoniae* has a great impact on amino acid synthesis^42^. Ultimately, the metabolites identified in the TB metabolic profile of our study are involved in pathways such as the oxidative metabolism of carbohydrates, proteins and fats, or the metabolism of the necessary amino acids and derivatives^41^.

When considering urine metabolomics, a study conducted in Uganda identified a urine-based metabolite biosignature with potential to monitor the response of individuals to anti-TB therapy^43^. In contrast, another study conducted in South Africa characterised the biological basis of a poor treatment outcome using urine samples collected at diagnosis, during treatment, and after treatment completion^44^. In a study of children and adults with TB, LTBI or uninfected, urine neopterin levels were measured using an enzyme-linked immunosorbent assay predicting that the neopterin/creatinine ratio may be a considerable predictor of disease progression^45^. According to our results, our models can detect TB even in patients who have initiated anti-TB treatment. In recent years, new markers have been identified in urine in response to TB treatment^43^. Future metabolomic studies should be conducted to monitor treatment and evaluate treatment or cure success, and also identify possible re-infections. In addition, host metabolites derived from anti-TB treatment identified in urine could be useful for monitoring treatment and improving patient adherence to TB treatment.

Limitations of this study include the relatively small sample size of TB patients before starting anti-TB treatment. Additionally, not all urine samples could be tested by HF and LF-NMR due to the time lapse between the analysis of the samples by the two NMR spectrometers. Therefore, some patients with samples tested for HF were not tested for LF-NMR and vice versa. Although this did not affect the results of the study, future research should test the discriminatory potential of the identified TB spectral fingerprint in a consecutive series of patients in a country with a high incidence of TB and co-infections. This proof of concept represents a first step towards the development of an affordable metabolomic test for the diagnosis of TB.

In this study, we have identified and characterised a metabolic profile for TB in urine with potential to discriminate TB patients from the rest of the study groups; this metabolic profile for TB has also been detected using an LF benchtop NMR spectrometer. The use of benchtop technology would facilitate its implementation in microscopy centres, health posts and primary care clinics, improving access to TB diagnosis. In addition, the ability of the model developed through this urine-based NMR technology to detect extrapulmonary TB and TB in patients under treatment is a step forward for the search for new diagnostics for TB that are not sputum-based and that can detect all forms of TB.

In summary, the identification of a metabolic profile for TB in urine from NMR with the potential to discriminate TB patients from pneumococcal pneumonia patients, individuals with LTBI, and uninfected individuals highlights the application of metabolomics as a new approach in the search of non-sputum-based new potential biomarkers for TB diagnosis.

## Methods

### Ethical statement

The study was approved by the ethical review board of the Ethics Committee of the HUGTiP and subsequently for all the Ethics Committee of all the health care centres participating (reference number CEI-PI-15-073). All patients gave written informed consent before being included. Sample collection and all experiments were performed in accordance with relevant guidelines and regulations.

### Study population

We have conducted a case–control study, in which we included patients with active TB, patients diagnosed with pneumococcal pneumonia, and healthy controls with latent TB infection (LTBI) and no infection. Participants were recruited through four different health care centres in Spain (Hospital Universitari Germans Trias i Pujol, Unitat de Tuberculosis de Drassanes de l'Hospital Universitari Vall d'Hebron, Serveis Clínics Unitat Clínica de Tractament Directament Observat de la Tuberculosi, and Hospital Sant Joan Despí Moisès Broggi), and one in Germany (Medical Clinic of the Research Center Borstel).

Participants were classified into four study groups: patients diagnosed with active TB, patients with pneumococcal pneumonia, individuals with LTBI, and uninfected individuals.

All active TB patients had clinical and radiological signs compatible with TB and were microbiologically confirmed by culture and/or Xpert MTB/RIF.

Patients with pneumococcal pneumonia were diagnosed by isolating the bacteria in blood culture and/or detecting the pneumococcal urinary antigen. Patients with pneumococcal pneumonia were collected before starting the antibiotic treatment.

LTBI individuals were recruited from contact tracing studies with a positive TST and/or IGRA but without clinical and radiological signs consistent with active TB. To perform the TST, we used a 2-TU dosage of PPD-RT (Statens Serum Institut, Copenhagen, Denmark). The performance and interpretation of the results of the Mantoux test were carried out following the Spanish guidelines^46^. IGRAs testing was performed using the commercially available enzyme-linked immunosorbent assay (ELISA) QuantiFERON-TB Gold In-Tube test (QFT, Qiagen, Hilden, Germany) and/or the Enzyme-Linked Immunospot (ELISPOT) assay T-SPOT.TB blood test (T-SPOT.TB; Oxford Immunotec Ltd, Oxford, UK) following the manufacturer’s protocol. The LTBI patients were included before starting chemoprophylaxis.

The uninfected individuals were volunteers with no evidence of *M. tuberculosis* infection and with negative TST and IGRA tests.

### Collection and preparation of urine samples for NMR analysis

We collected midstream urine samples from all participants of the study in sterile, universal plastic containers following standardised procedures^13^. Urine samples were aliquoted into 2 ml cryovials with screw caps and frozen at − 20 °C until the NMR experiments were performed. Before analysis, urine samples were thawed at room temperature and vortexed 30 s before use. We then aliquoted 400 µl of urine samples into Eppendorf tubes and added 250 µl of 0.2 M phosphate buffer solution containing 0.09% NaN_3_ to adjust the internal pH to 7.4. We adjusted the axis of chemical shifts to a signal reference at 0 ppm adding 0.3 mM trimethylsilyl propanoic acid (TSP) in deuterated water dissolution in the preparation of sample. Azide was added during the preparation of the urine samples to avoid bacterial contamination^14^. Buffered urines were vortexed for 30 s and centrifuged at 12,000*g* for 5 min. Then, we transferred 600 µl aliquot of the supernatant into 5 mm diameter NMR tubes (CortecNet, Les Ulis, France) for proton (^1^H) NMR acquisition. Figure 1 shows how many of these samples were analysed by HF and LF NMR.

### NMR spectral acquisition and processing

HF-NMR urine spectra were acquired using a Bruker 700 MHz NMR spectrometer (CNIO, Madrid, Spain) operating at a frequency of 697.87 MHz. Shimming and NMR preparation time was reduced to a minimum, while the sample for NMR analysis was chilled to 4 °C to minimise metabolic changes. The acquisition of the spectra was performed in accordance with the standardised protocols previously described^47^. A number of bidimensional homonuclear and heteronuclear experiments such as standard gradient-enhanced correlation spectroscopy (COSY), ^1^H–^1^H total correlated spectroscopy (TOCSY), and gradient-selected heteronuclear single quantum correlation (HSQC) protocols were performed to carry out component assignments. Between consecutive two-dimensional (2D) spectra, a control ^1^H NMR spectrum was always measured. No gross degradation was noted in the signals of multiple spectra acquired under the same conditions. Standard solvent-suppressed spectra were grouped into 32,000 data points, averaged over 256 acquisitions. The data acquisition lasted a total of 13 min using a sequence based on the first increment of the nuclear Overhauser effect spectroscopy (NOESY) pulse sequence to effect suppression of the water signal (δ =  ~ 4.80 ppm). Sample acquisitions were performed using a spectral width of 8333.33 Hz prior to Fourier transformation, and the free induction decay (FID) signals were multiplied by an exponential weight function corresponding to a line broadening of 0.3 Hz.

LF-NMR urine spectra were acquired using a Magritek Spinsolve 60 Ultra Benchtop spectrometer (Magritek GmbH, Aachen, Germany) at a frequency of 60 MHz using a one-dimensional presaturation (1D PRESAT) sequence to allow for efficient saturation of the water signal (δ =  ~ 4.95 ppm) following the previously described procedures^15^.

NMR data were processed and editing using MestReNova software (v.14; Mestrelab Research, Santiago de Compostela, Spain) according to the established protocols described in a previous study^47^. Metabolite signals of the spectra were shift-aligned using trimethylsilyl propanoic acid (TSP) as a reference signal standard (δ = 0.00 ppm). From the raw NMR spectra, the chemical shift region from 5.00 to 5.20 ppm was excluded from the analysis to remove the random effects of variation in the urine and water resonance suppression (δ = 6.50 to 4.22 ppm). Similarly, the chemical shift region from 0 to 0.04 ppm containing the internal reference (TSP) was excluded from the statistical analyses. Baseline correction was performed automatically using the ‘Withakker Smoother’ algorithm. Binning (also known as bucketing) was applied to NMR spectra and data-reduced to equal length integral segments (bins) of δ = 0.04 ppm to compensate variations in resonance positions. All bins were normalized by the total sum of the spectral regions (each bin was divided by the sum of all the NMR signals). Thus, the concentration of each metabolite was normalized by the urine concentration to compare these concentrations (in arbitrary units) between samples. Relative intensity was calculated as the original intensity normalized by total sum of the spectral regions to compensate urine concentration and to ensure that all observations were directly comparable.

### Data analysis

#### Statistical analysis of the study population

A descriptive analysis of the subjects who participated in the study was performed according to the study groups. Frequencies and percentages described the qualitative variables, while the mean and standard deviation described the quantitative variables. For comparisons between study groups, we used the chi-squared test in the case of qualitative variables, and the analysis of variance (ANOVA) in the case of quantitative variables. The level of significance was fixed at 0.05. Analyses were performed using the statistical software IBM SPSS Statistics v.25 (SPSS, Chicago, US).

#### Statistical analysis of metabolomic data

Data from ^1^H NMR spectra were analysed in a multivariate manner using the Metabonomic package of R software (rel.3.3.1)^48^. NMR spectra were data-reduced to equal length integral segments of δ = 0.04 ppm to compensate variations in resonance positions, and they were normalized by total sum of the spectral regions. Prior to multivariate statistical analysis, spectral data were Pareto scaled^49^. Unsupervised (blinded) data were analysed by PCA by the "prcomp" function from the statistical library and supervised (unblinded) analysis was by PLS-DA by the "gpls" function from the "gpls" package allowing separation between no more than two classes of samples.

PCA was applied to represent the variance of all metabolomic variables present in the data-reduced NMR spectra in a low-dimensional space (bins of δ = 0.04 ppm)^50^ to identify a differential metabolic pattern of TB to be used as potential biomarkers for TB diagnosis. Thus, all the spectral regions grouped in bins of δ = 0.04 ppm were transformed into a new set of orthogonal variables known as PCs. The first PC was defined by the spectral profile (load) in the data describing most of the variation; the second PC, was the second-best profile describing the variation, and so on, so that the retention of the variation present in the original variables decreased as we went down the order.

The PCs are composed of the scores and loadings. On one hand, the scores hold information about the samples (concentrations). Thus, PCA score plots of the first two or three PCs were used to visually observe the differences between the samples and immediately display sample clustering patterns according to their elemental composition^51,52^. In addition, PCA score plots were used to highlight statistical outliers. We use the Mahalanobis distance to confirm statistical outliers; this consists of calculating the distance from a data point to the centroid of all samples. Mahalanobis distance was calculated for PC1, PC2, and PC3. A single case was considered a statistical outlier if it was placed out of the tolerance ellipse of 97.5%^17^. On the other hand, the loadings hold information about the variables of the data set (chemical changes), indicating the importance of each region in explaining the variance between samples. Therefore, PCA loading plots were used to identify the multiple regions (δ = 0.04 ppm bins) of the ^1^H NMR spectra responsible for the separation between groups (the so-called metabolic fingerprint). The spectral regions (potential biomarkers for TB diagnosis) selected from PCA loading plots were confirmed by Hotteling’s T2 tests^18^. Those regions outside the 95% tolerance ellipse were identified as the spectral regions responsible for the metabolic differences in the PCA plots. Hotteling’s T2 test was applied for each PCA: (1) TB and uninfected groups, (2) TB and pneumococcal pneumonia groups, and (3) TB and LTBI groups.

The identification of the metabolites corresponding to the metabolic fingerprint was performed using the Human Metabolome Database^53^ and the characteristic cross-peaks from 2D HSQC spectra. The identified metabolites were individually integrated for metabolic quantification applying the Global Spectral Deconvolution analysis algorithm provided by the MestReNova software and corrected by the multiplicity of the NMR signal. Statistical significance was determined using a Bonferroni corrected Student's t-test assuming significant unequal corrected variance with p < 0.05^54^.

PLS-DA was applied by classifying patients into two groups^51^. We used the algorithm proposed by Ding and Gentleman et al.^55^ (tolerance for convergence: 1 × 10^–3^, the maximum number of iterations allowed: 100). The number of PLS components used was chosen by the percentage of variance explained, the R2, and the mean squared error of cross-validation graphics. Thus, PLS-DA was applied to the δ = 0.04 ppm bucketed NMR spectra of the following groups: TB vs. pneumococcal pneumonia, TB vs. LTBI, and TB vs. uninfected. PLS-DA predictive models were performed to assess and validate the diagnostic accuracy of the fingerprints of the metabolites present used to discriminate TB cases from the control groups. Before the comparison between groups (TB vs. pneumococcal pneumonia, TB vs. LTBI, and TB vs. uninfected), samples from each group were divided into two sets: the training set (50%) and the test set (50%). For training purposes, the classification functions derived from the probability of belonging to each group were computed with a number of random testing subjects. These classification functions were used afterwards to classify the rest of the subjects for internal validation. This process was repeated 100 times with random permutations of the training and test sets to reduce type I errors^55^. The percentages of correct classification were calculated as a measure of model performance.

## Supplementary Information

Supplementary Information.
