# Supplementary material for: Discovery and validation of an NMR-based metabolomic profile in urine as TB biomarker
Source: Sci Rep. 2020 Dec 18;10:22317. doi: 10.1038/s41598-020-78999-4 (PMC7749110; doi:10.1038/s41598-020-78999-4)
Supplement: Supplementary file 1 — Supplementary Information. [file 41598_2020_78999_MOESM1_ESM.docx]

**Title:** Discovery and validation of an NMR-based metabolomic profile in urine as TB biomarker

**Authors:**

José Luis Izquierdo-Garcia^1,2,3,+^, Patricia Comella-del-Barrio^2,4,5,+^, Ramón Campos-Olivas^6^, Raquel Villar-Hernández^2,4,5^, Cristina Prat-Aymerich^2,4,5,#a^, Maria Luiza De Souza-Galvão^7^, Maria Angeles Jiménez-Fuentes^7^, Juan Ruiz-Manzano^2,8^, Zoran Stojanovic^2,8^, Adela González^2,8^, Mar Serra-Vidal^4^, Esther García-García^4^, Beatriz Muriel-Moreno^4^, Joan Pau Millet^9,10^, Israel Molina-Pinargote^9^, Xavier Casas^9^, Javier Santiago^9^, Fina Sabriá^11^, Carmen Martos^11^, Christian Herzmann^12^, Jesús Ruiz-Cabello^1,2,3,13, ¶^, and José Domínguez^2,4,5, ¶,^*

**Affiliation:**

^1^ CIC biomaGUNE Center for Cooperative Research in Biomaterials, BRTA Basque Research and Technology Alliance, Donostia, Donostia, Gipuzkoa, Spain

^2^ CIBER de enfermedades respiratorias (CIBERES), Instituto de Salud Carlos III, Madrid, Spain

^3^ Universidad Complutense de Madrid. Facultad de Farmacia, Madrid, Spain

^4^ Servei de Microbiologia, Hospital Universitari Germans Trias i Pujol, Institut d’Investigació Germans Trias i Pujol, Badalona, Barcelona, Spain

^5^ Departament de Genètica i Microbiologia, Universitat Autònoma de Barcelona, Barcelona, Spain

^6^ CNIO Centro Nacional de Investigaciones Oncológicas, Madrid, Spain

^7^ Unitat de Tuberculosi de Drassanes, Servei de Pneumologia. Hospital Universitari Vall d’Hebron, Barcelona, Spain

^8^ Servei de Pneumologia, Hospital Universitari Germans Trias i Pujol, Barcelona, Spain

^9^ Serveis Clínics, Unitat Clínica de Tractament Directament Observat de la Tuberculosi, Barcelona, Spain

^10^ CIBER de Epidemiología y Salud Pública (CIBERESP), Instituto de Salud Carlos III, Madrid, Spain

^11^ Servei de Pneumologia, Hospital Sant Joan Despí Moises Broggi, Sant Joan Despi, Barcelona, Spain

^12^ Center for Clinical Studies, Research Center Borstel, Borstel, Germany

^13^ IKERBASQUE, Basque Foundation for Science, Bilbao, Vizcaya, Spain

^#a^ Current adress: Julius Centre for Health Sciences and Primary Care, University Medical Center Utrecht, Utrecht University, Utrecht, the Netherlands

*** Corresponding author:**

E-mail: [jadominguez@igtp.cat](mailto:jadominguez@igtp.cat) (JD)

**^+^** These authors contributed equally to this work.

^¶^ These authors also contributed equally to this work and as co-senior authorship.

**Supplementary Figures**


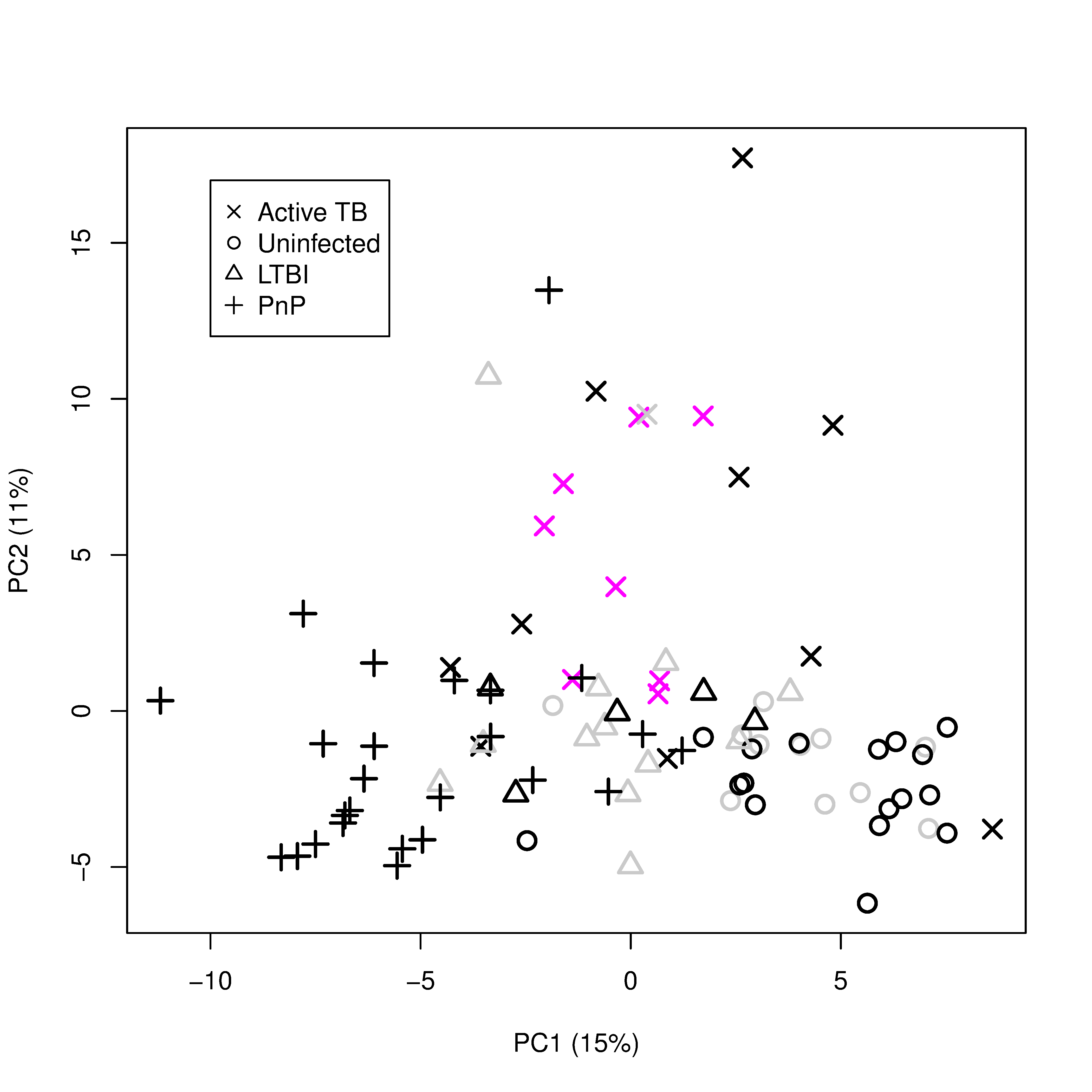


**Supplementary Figure S1.** Principal component analysis (PCA) score plots of urine spectra analysed by high-field nuclear magnetic resonance of untreated TB patients (n=19, x), uninfected individuals (n=28, circle ), pneumococcal pneumonia patients (n=25, cross) and LTBI individuals (n=17, triangle) colored according to the recruitment study centres in pink (Research Center Borstel), in grey (Unitat de Tuberculosi de Drassanes), and in black (Hospital Universitari Germans Trias i Pujol). ; TB, tuberculosis; PnP, pneumococcal pneumonia; LTBI: latent TB infection; PC, principal component.


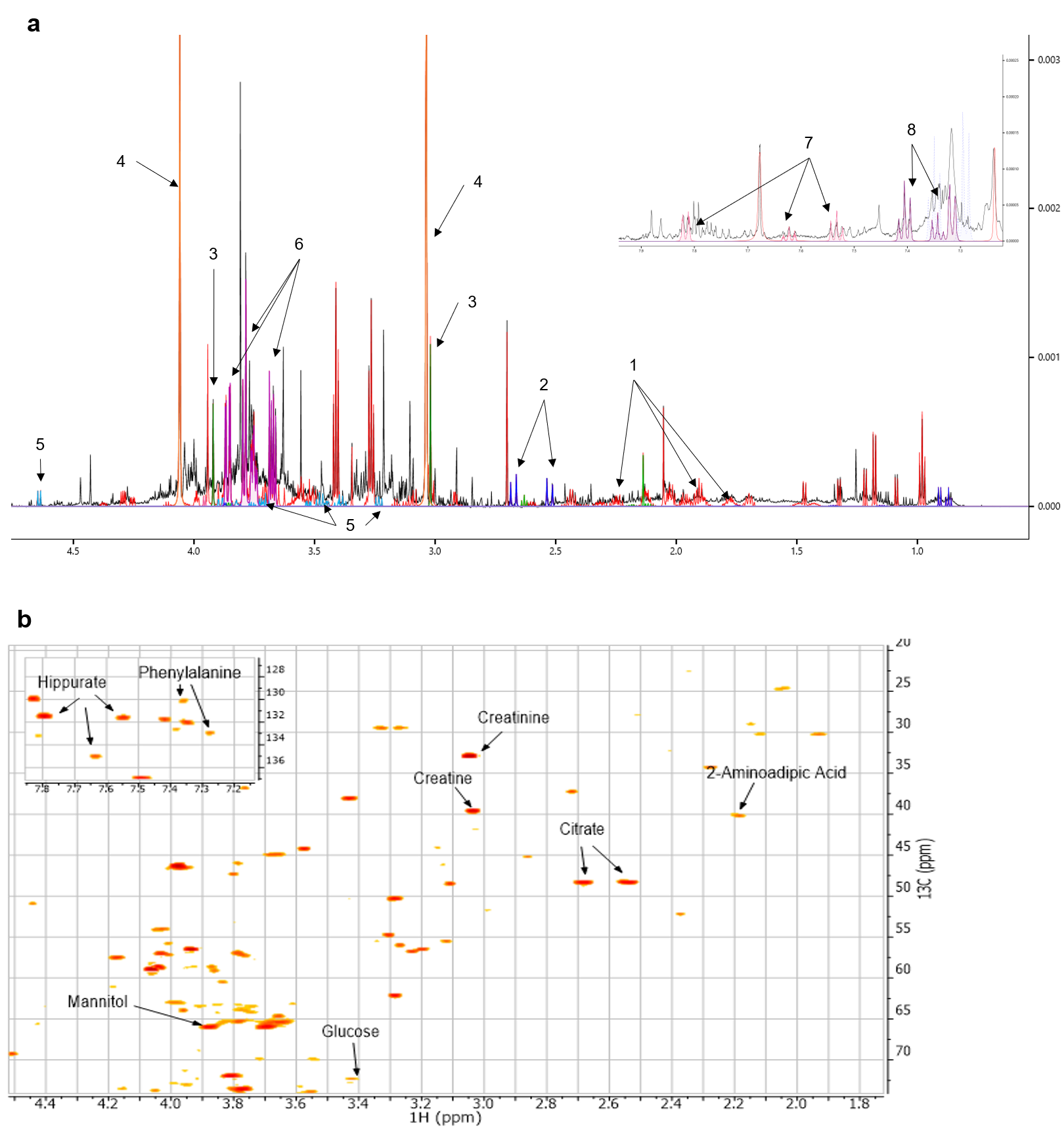


**Supplementary figure S2.** Metabolic assignments of the biomarkers responsible for the discrimination between TB patients and the rest of the control groups (A) Assignments based on cross-peaks from ^1^H-^13^C Heteronuclear Single Quantum Correlation Spectroscopy (HSQC) spectrum from a characteristic tuberculosis plasma sample. 1-Aminoadipic Acid, 2-Citrate, 3-Creatine, 4-Creatinine, 5-Glucose, 6-Mannitol, 7-Hippurate, 8-Phenylalanine. (B) PCA loading plot of the metabolic fingerprinting showing the eight metabolites.
